# Magnitude of Spousal Violence and Associated Factors among Midlife Women in Ethiopia

**DOI:** 10.4314/ejhs.v33i2.16

**Published:** 2023-03

**Authors:** Birye Dessalegn Mekonnen, Netsanet Balemual

**Affiliations:** 1 Amhara Public Health Institute, Central Gondar, Ethiopia; 2 Addis Ababa Health Bureau, Addis Ababa, Ethiopia

**Keywords:** Spousal violence, Factors, Midlife women, EDHS 2016, Ethiopia

## Abstract

**Background:**

Intimate partner violence is the most common form of gender-based violence and has enormous maternal health consequences. There is limited evidence concerning the magnitude and determinants of intimate partner violence amongst midlife women. Thus, this study aimed to determine the extent of and the factors contributing to spousal violence amongst midlife Ethiopian women.

**Methods:**

The 2016 Ethiopia Demographic and Health Survey (EDHS) data were examined, and a sample of 1628 ever-married midlife women was included. The analysis was performed using SPSS version 20. Bivariate and multivariable logistic regression analysis was conducted to examine the determinants of intimate partner violence. All statistical significance was declared at p value < 0.05.

**Results:**

The prevalence of spousal violence among midlife women in Ethiopia was 31.8%. Age of women, divorced, and working status were significantly associated with spousal violence. The likelihood of spousal violence is increase among midlife women who had no formal education, less decision-making power in household, and had partner who had drinking habit.

**Conclusion:**

This study showed that nearly one-thirds of midlife Ethiopian women have experienced spousal violence in their lifetime. Empowering midlife women by giving them decision-making tools and educating them to deal with, and prevent spousal violence may be effective strategies in reducing this problem.

## Introduction

Violence is an extreme form of aggression and a violation of fundamental human rights which is implicated in social, clinical health, and public health challenges (1–3). Although several interventions have been implemented to halt violence, it remains high among women and girls (2, 4). Intimate partner violence is defined as any type of behavior directed at either a woman or a girl by an intimate partner that causes physical, sexual, or psychological harm (2). Intimate partner violence is the most common form of gender-based violence and comprises all sexual, physical, or emotional harms as well as marital controlling behaviors by an intimate partner (5).

Domestic violence is prevalent among women and has been associated with poor reproductive health. A study conducted by the World Health Organization (WHO) revealed that the prevalence of lifetime spousal violence among ever-married women was 30% (2). Research have shown an increase in intimate partner violence in Sub-Saharan Africa (SSA) (6, 7). Furthermore, intimate partner violence in developing countries is higher than in developed countries, with a prevalence of almost 37% among reproductive age women (2). A systematic review and meta-analysis conducted in low and middle-income countries showed that the pooled prevalence of IPV was 38% within the last 12 months (8). In Ethiopia, spousal violence is still the highest contributor to gender based violence with about 34% of ever-married reproductive age women have experiencing spousal physical, sexual, or emotional violence in the 12 months preceding the 2016 Ethiopia Demographic and Health Survey (EDHS) (9).

Spousal violence has enormous maternal health consequence such as psychiatric illnesses, physical injuries, sexually transmitted infections, and unintended pregnancies which in turn may lead to forced and unsafe abortion and gynecological problems (10–13). Furthermore, research have provided a wide level of evidence suggesting that stillbirths, premature labour and low birth weight may be possible adverse effects of spousal violence (14,15).

The effects of spousal violence have been identified for women across the age continuum, with consequences remaining for a significant period after the violent experience has ended (16, 17). Midlife women who have experienced spousal violence over a long period of time may develop diminished self-esteem and self-worth (18). Mental health issues such as anxiety, depression, alcohol dependence, and poor family relationships may emerge in these women (19).

Recent exposure to violence in midlife women might also be associated with exposure to violence at different stages of their life-course (20, 21). There is limited understanding of help-seeking behaviors amongst midlife women who are subjected to violence, since ageing may influence a woman's decision to disclose or report abuse (22).

Spousal violence is an important public health concern and a priority issue on the United Nations Sustainable Development Goals (SDGs) agenda. Most studies using large scale surveys that focus on spousal violence among women of reproductive age group (15 to 49 years). Studies focusing on midlife women's experience of spousal violence are limited. Existing surveys and data on violence against women have focused on women of reproductive age (23–25), adolescents and young women (26, 27). Compared to adolescents and young women, midlife women may experience different relationship dynamics which influence types of violence (20, 28). Currently, existing evidence of quantitative data concerning spousal violence against midlife women is limited. Despite the national and international emphasis to reduce violence against women, the magnitude and determinants of spousal violence among midlife women is not well investigated in Ethiopia. Thus, it is crucial to determine the magnitude of spousal violence amongst midlife women in order to better identify determinants of this, and to plan targeted support services and interventions. This study aimed to address these gaps in the evidence, and to determine the magnitude and determinants of spousal violence amongst midlife Ethiopian women.

## Methods

**Data sources**: This population based cross-sectional study used secondary data from the 2016 EDHS. A two-stage cluster sampling was employed to obtain a nationally representative sample. The first and second stages involved the selection of 645 clusters (202 in urban and 443 in rural), and 28 households in each cluster, respectively.

The 2016 EDHS implemented a module of questions based on the most common form of violence against women which is domestic violence. As per the World Health Organization's (WHO) guidelines (29), in the 2016 EDHS only one eligible woman was randomly selected per household for interviewing, and the interview was not implemented if privacy could not be obtained. Accordingly, a total of 5,860 women were selected in the violence against women module (9). This analysis included all midlife women between 35–49 years of age, and who were married/in sexual partnerships. A total of 1628 (weighted) ever-married women were included in the final analysis. Data were weighted for the complex nature of the stratified, multistage cluster sampling strategy and for non-responses.

**Study variables**: The outcome variable was spousal violence where it combined all the three forms of violence (emotional, physical and sexual violence). Midlife women were asked independent questions indicating whether their husbands/partners had ever been, or were presently physically violent(hit, push, slap, kick, beat up, throw something; twist arm or pull hair; punch with fist or with something else; tries to choke or burn; threaten or attack with any material), sexual violence (force them to have sexual intercourse or perform any other sexual act against their will) and emotional violence (say something to humiliate them in front of others, insult them or make them feel bad, threaten to hurt them or someone they care about). The expected response was either ‘yes’ to any of the three questions and implied experience of any spousal violence and ‘no’ implied no experience of any spousal violence. The term spousal violence used interchangeably with intimate partner violence as the DHS data includes only ever-married women (30), which referring to both respondent's current and former spouses.

Based on the literature reviewed, the independent variables were age (35–39, 40–44, 45–49), education level of the women (No formal education, primary school, secondary school, and higher education), current marital status (married, divorced, widowed), religion (Orthodox, Muslim, Protestant and Catholic), residence (urban, rural), working status (yes, no), wealth index (poor, middle, rich), partner alcohol drinking habit (yes, no), frequency of listening radio (Not at all, ≤1 a week, > 1 a week), watching TV and reading newspaper (Not at all, ≤1 a week, > 1 a week), Reading newspaper (Not at all, ≤1 a week, > 1 a week), and Decision maker in household (Mainly respondent, mainly husband/partner, Jointly).

**Patient and public involvement**: Patients and members of the public were not directly involved in the design or planning and conduct of the study.

**Data processing and analysis**: The data were analyzed using SPSS version 20 statistical software packages. Frequencies and percentage of study variables were calculated to summarize selected background characteristics of women. Bivariate and multivariable logistic regression analysis was performed to identify the factors associated with intimate partner violence. Those determinant variables with p < 0.2 in the bivariate logistic analysis were included in the multivariate logistic regression analysis. Adjusted odds ratios (AOR) with 95% confidence interval (CI) were used to predict the strength of association between determinants and spousal violence. The model fitness was assessed using likelihood ratio test which shows the model was fitted, and multicollinearity between covariates was checked using the variance inflation factor (VIF) which showed VIF for each independent variable less than 10 (31). In all analyses, sampling weights that accounted for complex survey design were incorporated as per recommended. Variables that had a p value of <0.05 were considered statistically significant.

**Ethics Approval:** The protocol was approved by the Ethiopian Health and Nutrition Research Institute (EHNRI) Review Board, the National Research Ethics Review Committee (NRERC) at the Federal Democratic Republic of Ethiopia Ministry of Science and Technology, the ICF Macro Institutional Review Board, and the Centers for Disease Control and Prevention (CDC). As indicated in the EDHS 2016 publications, written consent for participation was obtained from each respondent. Though the dataset of the EDHS is not available as a public domain survey dataset, after developing protocol, the authors requested the data by registration on the MEASURE DHS website at: www.dhsprogram.com. Finally, access to the data for this research was granted from the demographic and health survey program team.

## Results

**Descriptive characteristics of study respondents**: A total of 1628 ever-married midlife women who reported their experience of intimate partner violence were included. The mean age and standard deviation of respondents was 40.62 ± 2.8 years. The majority (71.3%) of the midlives were married, resided in rural areas (71.5%) and had no formal education (43.2%). Regarding the wealth status of the women, about 49.3% of midlife women were from a poor family (Table 1).

**Table 1:** Socio-demographic and socio-economic characteristics of ever-married midlife women (N=1628) in Ethiopia, 2016

Variables	Frequency	Percent	Spousal violence		
			No (%)	Yes (%)	P-value
Age					
35–39	767	47.1	594 (77.4)	173 (22.6)	0.031
40–44	522	32.1	302 (57.9)	220 (42.1)	
45–49	339	20.8	215 (63.4)	124 (36.6)	
Marital status					
Married	1160	71.3	789 (68.1)	371 (31.9)	0.003
Divorced	412	25.3	272 (66.1)	140 (33.9)	
Widowed	56	3.4	50 (89.3)	6 (10.7)	
Religion					
Orthodox	1012	62.2	670 (66.2)	342 (33.8)	
Muslim	496	30.5	352 (71.0)	144 (29.0)	0.121
Protestant	79	4.4	54 (68.4)	25 (31.6)	
Catholic	41	2.5	35 (85.4)	6 (14.6)	
Place of residence					
Urban	464	28.5	251 (54.1)	213 (45.9)	0.001
Rural	1164	71.5	860 (73.9)	304 (26.1)	
Educational level					
No formal education	703	43.2	517 (73.5)	186 (26.5)	
Primary school	513	31.5	363 (70.8)	150 (29.2)	0.321
Secondary school	263	16.1	165 (62.7)	98 (37.3)	
Higher education	149	9.2	66 (44.3)	83 (55.7)	
Current working status					
Yes	613	37.7	384 (62.6)	229 (37.4)	0.041
No	1015	62.3	727 (71.6)	288 (28.4)	
Wealth status					
Poor	803	49.3	595 (74.1)	208 (25.9)	0.217
Middle	547	33.6	334 (61.1)	213 (38.9)	
Rich	278	17.1	182 (65.5)	96 (34.5)	
Watching TV					
Not at all	1055	64.8	663 (62.8)	392 (37.2)	0.462
.1 a week	248	15.2	132 (53.2)	116 (46.8)	
> 1 a week	325	20.0	316 (97.2)	9 (2.8)	
Listening Radio					
Not at all	1039	63.8	654 (62.9)	385 (37.1)	
.1 a week	214	13.1	126 (58.9)	88 (41.1)	0.318
> 1 a week	375	23.1	331 (88.3)	44 (11.7)	
Reading newspaper					
Not at all	1455	89.4	980 (67.4)	475 (32.6)	0.112
.1 a week	57	3.5	41 (71.9)	16 (28.1)	
> 1 a week	116	7.1	90 (77.6)	26 (22.4)	
Decision maker in household					
Mainly respondent	376	23.1	289 (76.9)	87 (23.1)	0.014
Mainly husband/partner	825	50.7	727 (88.1)	98 (11.9)	
Jointly	427	26.2	95 (22.2)	332 (77.8)	
Husband drinks alcohol					
Yes	121	7.4	67 (55.4)	54 (44.6)	0.321
No	1507	92.6	1044 (69.3)	463 (30.7)	

**Figure 2:**
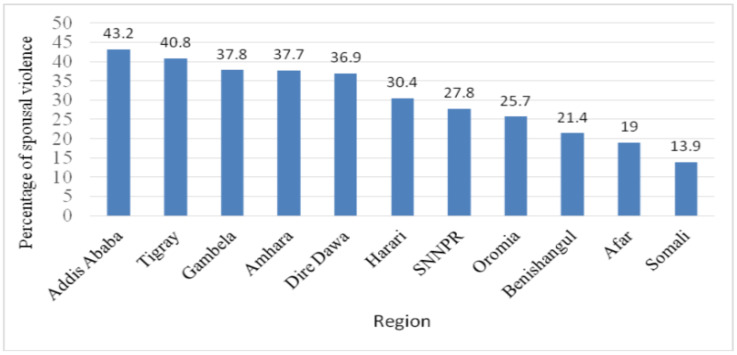
Percentage of midlife women who have experienced spousal violence by region of Ethiopia, 2016.

**Prevalence of spousal violence among midlife women**: The lifetime prevalence of intimate partner violence among ever married midlife women in Ethiopia was 31.8% (95% CI: 30.6, 33.2). Of this, the prevalence of physical, sexual and psychological violence was 21.2%, 18.4%, and 16.1% respectively. The maximum spousal violence against midlife women is found in Addis Ababa city administration (43.2%) while the lowest (13.9%) is observed in Somali region (Figure 2).

**Factors associated with spousal violence**: In multivariable logistic regressions analysis; age of women, current marital status, educational status of women, working status of women, partner alcohol drinking habit and decision maker in household were associated with spousal violence against midlife women. Midlife women aged between 45–49 years were more likely (AOR = 1.94; 95% CI: 1.19, 4.43) to experience spousal violence as compared to those whose age between 35–39 years. The likelihood of experiencing spousal violence for divorced midlife women was 1.71 times more likely (AOR = 1.71; 95% C.I: 1.31, 2.21) compared to currently married midlife women. The likelihood of experiencing spousal violence was less likely among midlife women with primary education (AOR = 0.70; 95% CI: 0.59, 0.84), secondary education (AOR = 0.73; 95% CI: 0.57, 0.94) and higher education (AOR = 0.62; 95% CI: 0.45, 0.85) compared to those midlife women with no education. Midlife women who were employed (working status) had lower odds (AOR = 0.77; 95% CI: 0.60–0.99) of experiencing spousal violence compared to those midlife women who were not working.

Furthermore, alcohol drinking habits of midlife women's partner was also associated with spousal violence. Midlife women who had a husbands/partner who had drinking habits had higher odds of experiencing spousal violence (AOR = 3.66; 95% CI: 2.88, 4.64) compared to those whose partners were never drunk. Moreover, midlife women whose husbands/partners made decision in household mainly had higher odds of experiencing intimate partner violence spousal violence (AOR = 9.29; 95% CI: 6.63, 13.03) compared to those who made a joint decision within the couple (Table 2).

**Table 2:** Bivariate and multivariable logistic regression analysis of factor associated with spousal violence among midlife women in Ethiopia, 2016

Variables	COR (95% CI)	p-value	AOR (95% CI)	p-value
Age				
35–39	1		1	
40–44	1.08(0.90, 2.68)	0.081	1.11(0.94, 3.08)	0.076
45–49	1.46(1.24, 3.42)	0.004	1.94(1.19, 4.43)*	0.003
Marital status				
Married	1		1	
Divorced	1.09(0.91, 1.31)	0.092	1.71(1.31, 2.21)*	0.002
Widowed	0.76(0.51, 1.14)	0.422	0.93(0.57, 1.53)	0.413
Residence				
Urban	1.14(0.99, 1.31)*	0.332	1.11(0.92, 1.56)	0.341
Rural	1		1	
Educational status				
No formal education	1		1	
Primary school	0.68(0.59, 0.78)*	0.001	0.70(0.59, 0.84)*	0.001
Secondary school	0.72(0.59, 0.87)*	0.001	0.73(0.57, 0.94)*	0.001
Higher education	0.73(0.57, 0.94)*	0.002	0.62(0.45, 0.85)*	0.001
Wealth status				
Poor	0.91(0.79, 1.04)	0.463	0.86(0.72, 1.02)	0.362
Middle	1.06(0.89, 1.26)	0.181	0.79(0.66, 1.97)	0.336
Rich	1		1	
Respondents working status				
Yes	0.70(0.62, 0.79)*	0.882	0.70(0.59, 0.82)*	0.674
No	1		1	
Listening Radio				
Not at all	1		1	
.1 a week	1.01(0.79, 2.47)*	0.761	1.79(0.98, 4.81)	0.841
> 1 a week	1.12(0.81, 1.71)	0.285	1.81(0.93, 4.09)	0.249
Reading newspaper				
Not at all	1		1	
≥1 a week	0.57(0.25, 1.09)	0.087	1.32(0.92, 2.64)	0.138
≤ 1 a week	0.41(0.35, 1.54)	0.654	1.19(0.86, 1.65)	0.496
Decision maker in household				
Mainly respondent	1.33(0.94, 5.31)	0.098	1.45(0.87, 5.38)	0.098
Mainly husband/partner	2.57(2.21, 3.01)*	0.001	9.29(6.63, 13.03)*	0.001
Joint decision	1		1	
Husband drinks alcohol			
Yes	4.03(3.27, 4.96)*	0.001	3.66(2.88, 4.64)*	0.001
No	1		1	

* Statistically significant (p value <0.05)

## Discussion

This study analysed data from the 2016 Ethiopian DHS to examine the prevalence of spousal violence against midlife women. The study revealed that nearly one-thirds (31.8%) of midlife women reported having ever experienced spousal violence. This finding indicates that a substantial number of midlife women in the country are experiencing spousal violence. Furthermore, this finding suggests that there is a need to evaluate existing interventional programs, and to design evidence-based strategies that will respond to and prevent spousal violence amongst midlife women.

The prevalence of spousal violence against midlife women in this study is comparable with the result of other similar studies in Turkey (30.0%) (32) and the Ivory Coast (32.1%) (33). This implies that spousal violence amongst midlife women is a global public health concern. However, the prevalence seen in this study was relatively low compared to a finding from low and middle income countries where the prevalence was 37% (2). Moreover, this result was lower than other similar studies conducted in Kenya (34), Uganda (35), and Ghana (39%) (36). The reason for this variation may be due to differences in culture, belief, norms and traditions across regions, even though nationwide. The other reason could be due differences in the likelihood of reporting intimate partner violence experienced in midlife women.

The highest spousal violence against midlife women is found in Addis Ababa city administration (43.2%) while the lowest (13.9%) is observed in Somali region. The possible reason could be women in Somali region may not disclosure their experience of spousal violence due to cultural norms whereby women generally expected to be subordinate to men. This makes the women accept violence as a normal condition (37, 38).

Midlife women aged between 45–49 years were more likely to experience spousal violence as compared to those aged between 35–39 years. This finding is consistent with a Turkish study (32) and an Australian study (39), which suggests that menopausal symptoms may provoke conflict between couples when a woman became irritable and develops mood changes during the menopausal transition (40).

Marital status was associated with spousal violence amongst midlife women. Divorced women were more likely to experience spousal violence compared to currently married midlife women. This finding is supported by a study conducted in Arkansas and New Mexico (41), and may be due to the fact that married midlife women are generally more likely to compromise on certain issues causing less conflict in their homes.

Midlife women's educational status was significantly associated with spousal violence as midlife women with primary, secondary, or higher education had decreased odds of experiencing spousal violence compared to those with no education. This could be attributed to the fact that education may enable midlife women to get information about their human rights status and allow them to have better negotiating ability with their partners, helping to change male-controlled norms and values (42).

Working status was significantly associated with spousal violence amongst midlife women. Midlife women who were working had lower odds of experiencing spousal violence compared to those midlife women who were not working. This indicated that women who work may contribute financially to the household economy, and are thus involved in the decision-making process of household issues, which means that they may have a lower chance of experiencing spousal violence.

Midlife women with partners who drink alcohol were more likely to experience spousal violence when compared to their counterparts, whose partners did not consume alcohol. This finding is confirmed by other studies in Uganda (35), Ghana (36), and Ethiopia (43). This may be explained by the fact that alcohol can cause irresponsible behaviours, aggression, an altered mental state and clouded judgment, which may increase the likelihood of a violent attack (44).

The study found that midlife women with low decision-making power in household issues were more likely to have experienced spousal violence than those who had a joint decision-making arrangement. This finding is similar to data from a study conducted in Bangladesh (45). This may be a result of cultural norms whereby women generally expected to be subordinate to men and do not make joint decisions regarding household issues. Moreover, midlife women with low decision-making power in household issues may not be more empowered to fight for their rights and make certain independent decisions.

This study could not determine causality among the key variables as the data were cross-sectional. Furthermore, the self-reporting of spousal violence is associated with underreporting and social desirability biases. Subsequently, midlife women may have been hesitant to disclose their experiences of spousal violence, which may affect the reported prevalence in this study. Furthermore, the study does not include older women in midlife (50–60 years) as the EDHS included only women of reproductive age group (15 to 49 years). Moreover, community-related factors were not assessed, due to a lack of information in the dataset. Apart from these limitations, this study provides a robust examination of spousal violence amongst midlife women using a nationally representative sample.

This study showed that nearly one-thirds of ever-married midlife women have ever experienced spousal violence in their lifetime. Age of women, being divorced, educational level of women, working status of women, partner alcohol drinking habit and low decision-making power in the household were found to be significant predictors of spousal violence. Thus, policy-makers, public health experts, programmers and interested stakeholders should establish effective strategies to minimize the problem of spousal violence and identifiable risk factors. Moreover, empowering women in decision-making and improving educational level may be effective strategies in reducing spousal violence against midlife women.

## Figures and Tables

**Figure 1:**
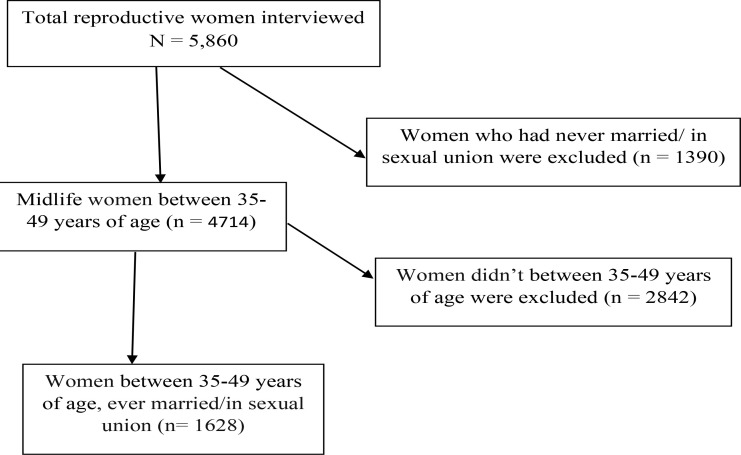
The diagrammatic presentation of stages of sampling.
